# Resveratrol Prevents Cardiovascular Complications in the SHR/STZ Rat by Reductions in Oxidative Stress and Inflammation

**DOI:** 10.1155/2015/918123

**Published:** 2015-02-23

**Authors:** Rebecca K. Vella, Candice Pullen, Fiona R. Coulson, Andrew S. Fenning

**Affiliations:** School of Medical and Applied Sciences, Central Queensland University, Building 81, Bruce Highway, Rockhampton, QLD 4701, Australia

## Abstract

The cardioprotective effects of resveratrol are well established in animal models of metabolic disease but are yet to be investigated in a combined model of hypertension and diabetes. This study investigated the ability of resveratrol's antioxidant and anti-inflammatory effects to prevent cardiovascular complications in the spontaneously hypertensive streptozotocin-induced diabetic rat. Diabetes was induced in eight-week-old male spontaneously hypertensive rats via a single intravenous injection of streptozotocin. Following this, resveratrol was administered orally for an eight-week period until the animals were sixteen weeks of age. Upon completion of the treatment regime assessments of oxidative stress, lipid peroxidation, inflammation, and cardiovascular function were made. Resveratrol administration to hypertensive-diabetic animals did not impact upon blood glucose or haemodynamics but significantly reduced oxidative stress, lipid peroxidation, and inflammatory cytokines. Reductions in systemic levels of oxidative stress and inflammation conferred improvements in vascular reactivity and left ventricular pump function and electrophysiology. This study demonstrates that resveratrol administration to hypertensive diabetic animals can elicit cardioprotective properties via antioxidant and anti-inflammatory effects. The observed preservation of cardiovascular function was independent of changes in blood glucose concentration and haemodynamics, suggesting that oxidative stress and inflammation are key components within the pathological cascade associated with hypertension and diabetes.

## 1. Introduction

Changes in dietary habits and lifestyle choices over recent decades have led to the increased prevalence of metabolic disorders with the World Health Organization estimating that 347 million people worldwide suffer from diabetes and 1 in 3 adults has hypertension [1]. Naturally derived compounds such as resveratrol are being explored as potential avenues of treatment and mechanistic drug design for cardiovascular disorders.

There are numerous pharmacological effects that have been attributed to resveratrol, including anti-inflammatory, antioxidant, antifungal, and anticancer properties [2]. Resveratrol has also been reported to induce relaxation of vascular tissues in both endothelium-dependent manner and endothelium-independent manner by preventing vascular remodelling and enhancing endothelial NO production [3, 4]. In rat models of hypertension, resveratrol has been found to act through antioxidant and anti-inflammatory mechanisms to prevent vascular and left ventricular remodelling and dysfunction [5], whilst in diabetic rodents resveratrol alleviated cardiac dysfunction by upregulating the nitric oxide-thioredoxin-heme oxygenase-vascular endothelial growth factor system, leading to an increase in manganese superoxide dismutase [6].

Spontaneously hypertensive streptozotocin-induced diabetic (SHR/STZ) rats are a reliable animal model for studying the synergistic effects of both hypertension and diabetes [7] as they develop significant elevations in plasma glucose levels, polyuria, albuminuria, and glucosuria and a pronounced loss of abdominal adipose tissue and body weight together with severe hypertension [8, 9]. Reports indicate that this induces a more severe cardiac functional deficit than either disease alone and can be described as a greater reduction in cardiac performance, exacerbated fibrosis, and hypertrophy that are associated with a relatively greater increase in oxidative stress and upregulation of the renin-angiotensinogen-aldosterone system [7, 10–12]. Similar results have been observed in humans in the fact that fibrosis and cardiac hypertrophy are more pronounced in those who suffer from both hypertension and diabetes as opposed to one disorder in isolation [13].

The aim of this study was twofold: firstly, to assess resveratrol's ability to prevent cardiovascular complications associated with diabetes and hypertension, independent of changes in haemodynamics and blood glucose levels using the SHR/STZ rat model, and, secondly, to determine if these effects are mediated through antioxidant and anti-inflammatory mechanisms.

## 2. Methods

### 2.1. Experimental Animals

Male Wistar-Kyoto (WKY) and spontaneously hypertensive rats (SHR) used in this study were randomly assigned to one of four groups, control (WKY, *n* = 20), resveratrol treated control (WKY + Res, *n* = 20), hypertensive-diabetic (SHR/STZ, *n* = 20), and resveratrol treated hypertensive-diabetic (SHR/STZ + Res, *n* = 20). Sample size was determined using a power analysis, ensuring that statistically meaningful results were obtained and unnecessary use of animals was avoided. This study was conducted over an 8-week period, at the end of which, animals were euthanized with pentobarbitone (187.5 mg/mL). During the treatment period, animals were housed on a 12-hour light/darkness cycle and given unlimited access to water and chow pellets. Ethical clearance for this project was obtained from the Animal Ethics Committee of Central Queensland University under guidelines from the National Medical Research Council of Australia.

### 2.2. Induction of Diabetes and Resveratrol Treatment Regime

Diabetes was induced in 8-week-old SHRs by a single intravenous dose of streptozotocin (55 mg/kg) into the femoral vein. Animals displaying symptoms of hyperglycemia (>15 mmol/L), polyuria, and a failure to thrive were considered diabetic. Insulin was not administered to any animals during this study. Administration of resveratrol (2 mg/kg/day via oral gavage) commenced when animals were 8 weeks of age and continued for a period of 8 weeks. This dose was selected for its minimal effects on systolic blood pressure and blood glucose levels, allowing for the suggestions that any cardioprotective effects observed could be attributed to the attenuation of underlying pathological mechanisms.

### 2.3. Physiological Parameters

Body weight and water consumption were assessed weekly for the duration of the study. 24 hours before euthanasia, animals were sedated with i.p. Zoletil (tiletamine 15 mg/kg with zolazepam 15 mg/kg) and assessments of systolic blood pressure and heart rate were made via tail cuff plethysmography. At the time of euthanasia, blood glucose levels (mmol/L) and wet weights of left ventricles, right ventricles, and kidneys, normalised to body weight, were recorded.

### 2.4. Biochemical Assessment of Nitric Oxide, Lipid Peroxidation, and Inflammation

Serum levels of total nitric oxide were assessed using a Griess reaction, where nitrate was reduced to nitrite in the presence of copper coated cadmium pellets, followed by colorimetric assessment [14]. Serum samples were assessed in duplicate for the concentration of malondialdehyde [15]. Briefly, samples were deproteinised, derivatised, and purified before undergoing HPLC assessment (C18 column, 4.6 × 150 mm length, injection volume 90 *μ*L, mobile phase was a mixture of 0.02% acetic acid (60%) and acetonitrile (40%), flow rate of 1.4 mL/min, detection wavelength 310 nm, and reference wavelength 360 nm). Methyl-malondialdehyde standards for this procedure were produced in accordance with Sim et al. [16]. Assessments of sera interleukin-1*β* and interleukin-6 were made through the use of R&D Systems DuoSet ELISA Development Systems which provide the immunological components required to perform a sandwich ELISA. Colorimetric assessment was made through the use of streptavidin-horse radish peroxidase with the detection wavelength of 450 nm (wavelength correction at 540 nm).

### 2.5. Conduit and Resistant Arteries

Thoracic aorta and second order mesenteric arteries were suspended within organ baths containing gassed (O_2_ (95%)/CO_2_ (5%)) Tyrode's solution (all in mM concentrations: NaCl 136.9, KCl 5.4, MgCl_2_ 1.05, NaH_2_PO_4_ 0.42, NaHCO_3_ 22.6, CaCl_2_ 1.8, glucose 5.5, ascorbic acid 0.28, and EDTA 0.1) at 37°C and allowed to equilibrate for a thirty-minute period. Following this, aortic rings and mesenteric arteries were loaded with a preset resting tension of 10 mN and 2 mN, respectively, and cumulative concentration response curves were performed using noradrenaline, acetylcholine, and sodium nitroprusside. Any fluctuations to the preset tension after the addition of vasoactive agents were recorded using PowerLab data acquisition units and chart software [17–19].

### 2.6. Isolated Langendorff Heart Preparation

The nonrecirculating heart preparation was used for isolated myocardial experiments. Briefly, hearts were excised and the aorta was cannulated via the dorsal root, allowing for retrograde perfusion with gassed (O_2_ (95%)/CO_2_ (5%)) Krebs-Henseleit Buffer (KHB) (all in mM concentrations: NaCl 119.1, KCl 4.75, MgSO_4_ 1.19, KH_2_PO_4_ 1.19, NaHCO_3_ 25.0, glucose 11.0, and CaCl_2_ 2.16) at a constant pressure of 100 mmHg and temperature of 37°C. A latex balloon catheter connected to a pressure transducer was then inserted into the left ventricle and hearts were atrially paced at 250 bpm. After an equilibration period, measurements of end-diastolic pressure were recorded in 5 mmHg increments (beginning at 0 mmHg up to 30 mmHg), allowing for the assessment of diastolic stiffness. Maximal rates of contraction and relaxation were calculated at a load of 10 mmHg of pressure [17].

### 2.7. Left Ventricular Microelectrode Studies

Cardiac electrophysiological changes were assessed in accordance with Fenning et al. [17], whereby left ventricular papillary muscles were suspended in an experimental chamber containing gassed (O_2_ (95%)/CO_2_ (5%)) Tyrode's solution warmed to 35°C. Within the experimental chamber, the muscle was positioned between two platinum electrodes, connected to a bioamplifier and stretched to a maximum preload (5 mN). Field stimulation at a frequency of 1 Hz, a pulse width of 0.5 msec, and stimulus strength 20% above threshold was commenced. Following a ten-minute equilibration period, intracellular bioelectrical activity was recorded. Parameters assessed include action potential duration at 20%, 50%, and 90% repolarisation, resting membrane potential, and force of contraction.

### 2.8. Data Analysis

Results were analysed using two-way analysis of variance with a Bonferroni post hoc test and Student's *t*-test where appropriate. Significance was determined to be *P* < 0.05, ^#^WKY versus SHR/STZ, ^†^SHR/STZ versus SHR/STZ + Res. All data is presented as mean ± S.E.M.

### 2.9. Drugs and Chemicals

Resveratrol, streptozotocin, noradrenaline, acetylcholine, and sodium nitroprusside were purchased from the Sigma Chemical Company, St. Louis, MO, USA. Serial dilutions of noradrenaline, acetylcholine, and sodium nitroprusside were dissolved in distilled water; resveratrol was suspended in a solution of nanopure water and DMSO to derive a preparation with a concentration of 2 mg/mL, and streptozotocin was suspended in citrate buffer immediately before injection. Reagents and chemicals utilised in the biochemical assays were of analytical grade and were purchased from the Sigma Chemical Company (St. Louis, MO, USA) and ThermoFisher Scientific (Australia).

## 3. Results

### 3.1. Physiological Parameters

**Figure 1:**
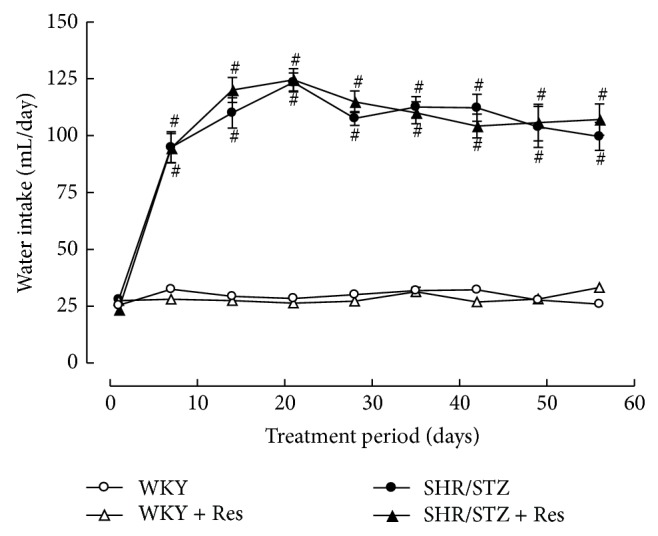
Weekly water consumption for WKY, WKY + Res, SHR/STZ, and SHR/STZ + Res treated rats. ^#^ *P* < 0.05 versus WKY.

**Figure 2:**
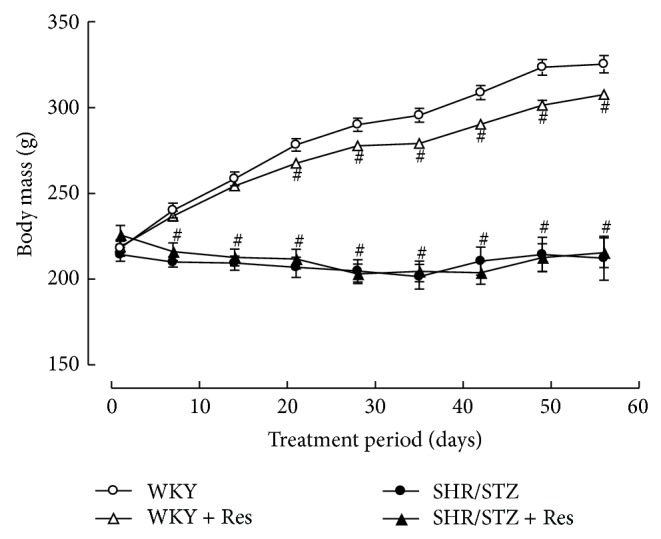
Weekly body weight for WKY, WKY + Res, SHR/STZ, and SHR/STZ + Res treated rats. ^#^ *P* < 0.05 versus WKY.

The induction of diabetes in SHR/STZ animals was confirmed via significant increases in blood glucose levels, excessive water consumption, and their inability to gain weight (Figures 1 and 2; Table 1). Consistent with the development of hypertension, SHR/STZ animals had a significant increase in systolic blood pressure. Eight weeks of untreated diabetes in this animal model also led to a significant reduction in heart rate (Table 1). Hypertrophy of the left and right ventricles and kidneys was also observed in SHR/STZ animals (Table 1). Resveratrol administration to SHR/STZ animals did not significantly impact upon blood glucose levels, water consumption, body weight, systolic blood pressure, heart rate, or systemic organ hypertrophy (Figures 1 and 2; Table 1).

### 3.2. Biochemical Assessments of Nitric Oxide, Lipid Peroxidation, and Inflammation

In comparison to WKY control animals, hypertensive-diabetic rats had a significant reduction in serum nitric oxide levels (30% reduction) and significant increases in serum malondialdehyde (106% increase), IL-1*β* (27% increase), and IL-6 (10% increase) concentrations (Table 1). Attenuation of decreased serum nitric oxide availability, increased malondialdehyde, and increased IL-1*β* levels were observed in resveratrol treated SHR/SRZ animals (Table 1).

### 3.3. Vascular Reactivity in Isolated Thoracic Aortic Rings

**Figure 3:**
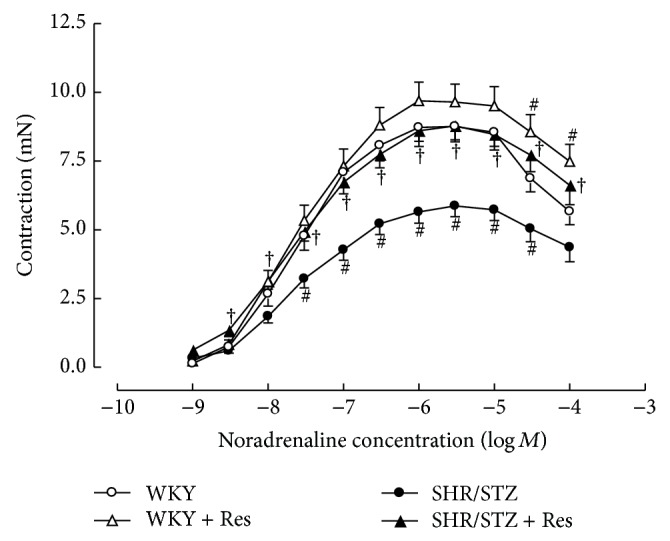
Cumulative concentration response to noradrenaline in isolated thoracic aortic rings from WKY, WKY + Res, SHR/STZ, and SHR/STZ + Res treated rats. ^#^ *P* < 0.05 versus WKY, ^†^ *P* < 0.05 versus SHR/STZ.

**Figure 4:**
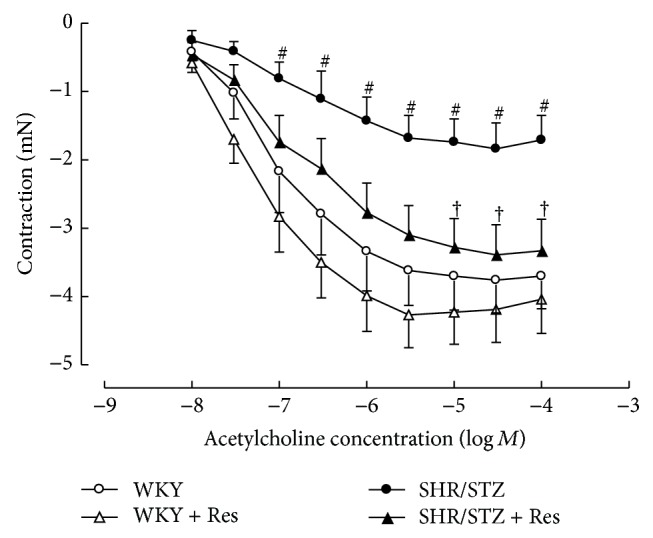
Cumulative concentration response to acetylcholine of noradrenaline precontracted thoracic aortic preparations from WKY, WKY + Res, SHR/STZ, and SHR/STZ + Res treated animals. ^#^ *P* < 0.05 versus WKY, ^†^ *P* < 0.05 versus SHR/STZ.

**Figure 5:**
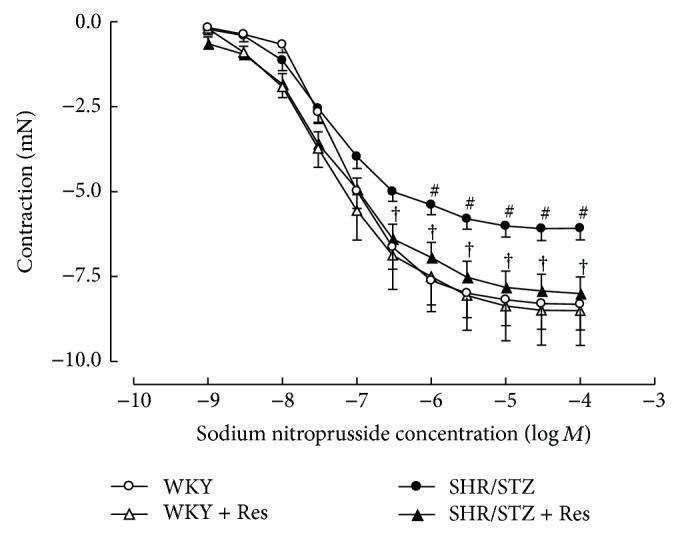
Cumulative concentration response to sodium nitroprusside of noradrenaline precontracted thoracic aortic preparations from WKY, WKY + Res, SHR/STZ, and SHR/STZ + Res treated rats. ^#^ *P* < 0.05 versus WKY, ^†^ *P* < 0.05 versus SHR/STZ.

Aortic tissue from SHR/STZ animals had significantly impaired adrenergic-mediated contractile response and endothelial-independent and endothelial-dependent relaxation responses (Figures 3, 4, and 5). Resveratrol administration to SHR/STZ animals restored smooth muscle-mediated contractions and relaxations in aortic tissue to levels observed in the WKY control animals (Figures 3 and 4).

### 3.4. Vascular Reactivity in Isolated Mesenteric Vessels

**Figure 6:**
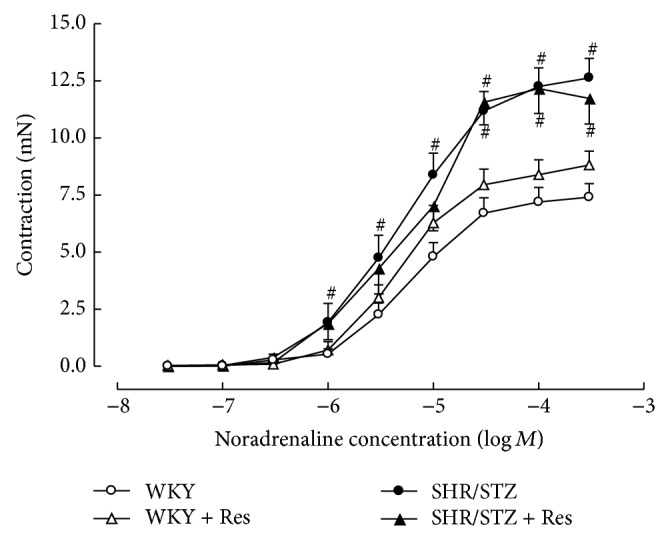
Cumulative concentration response to noradrenaline in isolated mesenteric arteries from WKY, WKY + Res, SHR/STZ, and SHR/STZ + Res treated rats. ^#^ *P* < 0.05 versus WKY.

**Figure 7:**
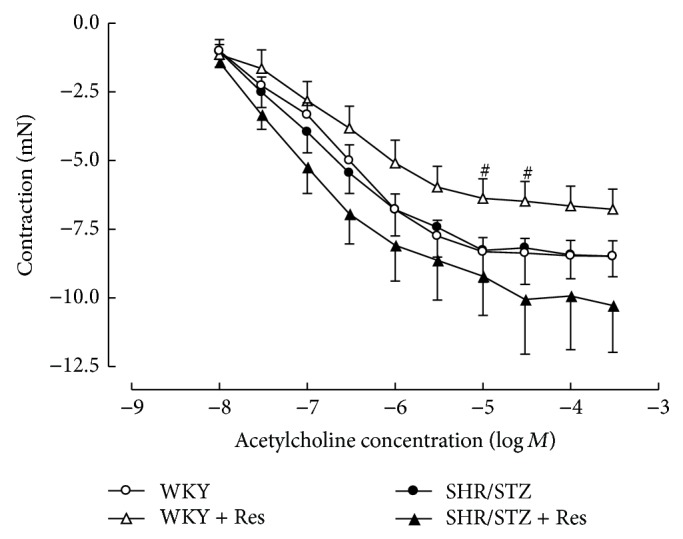
Cumulative concentration response to acetylcholine of noradrenaline precontracted mesenteric arteries from WKY, WKY + Res, SHR/STZ, and SHR/STZ + Res treated rats. ^#^ *P* < 0.05 versus WKY.

**Figure 8:**
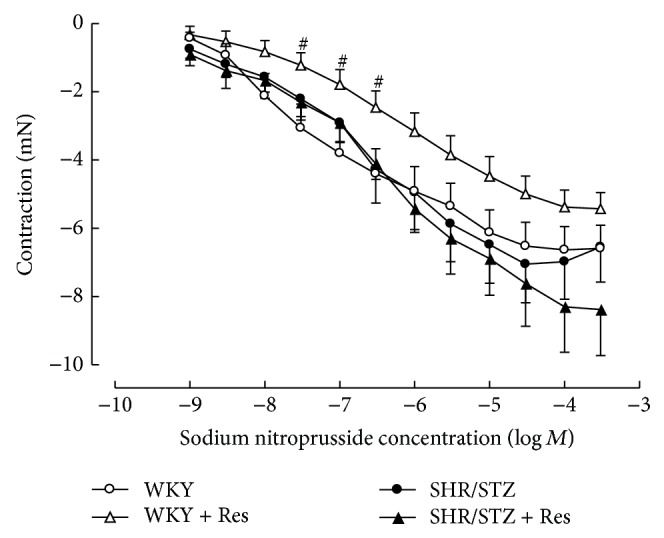
Cumulative concentration response to sodium nitroprusside of noradrenaline precontracted mesenteric arteries from WKY, WKY + Res, SHR/STZ, and SHR/STZ + Res treated rats. ^#^ *P* < 0.05 versus WKY.

Mesenteric vessels from SHR/STZ animals produced an increased contractile response to noradrenaline that was not altered by resveratrol administration (Figure 6). Neither the onset of hypertension/diabetes nor the administration of resveratrol impacted upon endothelium-independent or endothelium-dependent relaxation responses in resistance arteries (Figures 7 and 8).

### 3.5. *Ex Vivo* Left Ventricular Function

Compared to WKY control animals, the SHR/STZ hearts showed significant decreases in left ventricular compliance, developed pressure, and maximal rates of contraction and relaxation (Table 2). Resveratrol administration to SHR/STZ animals normalised left ventricular compliance and maximal rates of contraction and relaxation but did not significantly impact upon developed pressure (Table 2).

### 3.6. Left Ventricular Electrophysiology

Left ventricular papillary muscles isolated from SHR/STZ animals had significantly prolonged cardiac action potential duration at 20%, 50%, and 90% of repolarisation, which was normalised by resveratrol therapy (Table 2). Neither the onset of hypertension/diabetes nor the administration of resveratrol significantly impacted upon resting membrane potential or force of contraction (Table 2).

## 4. Discussion and Conclusions

Key observations from this study were as follows: (1) SHR/STZ animals had significant changes in oxidative stress, lipid peroxidation, and inflammatory status that were accompanied by dysfunction of the heart and blood vessels and (2) resveratrol administration to SHR/STZ animals elicited cardioprotective effects, independent of alterations in blood glucose levels or haemodynamics, that were attributed to antioxidant and anti-inflammatory mechanisms. These results indicate that, in a genetic model of hypertension superimposed with type I diabetes, changes to redox and inflammatory status are equally important in the development of cardiovascular dysfunction as alterations in metabolism and haemodynamics thus supporting results from Huynh et al. [20]. Additionally it is well established that diabetes and hypertension present concomitantly and often act synergistically to promote the pathological state via the induction of oxidative stress and inflammation [10, 21–23]. Consistent with these observations, SHR/STZ animals within the current study had reduced cardiovascular function that was attributed to systemic increases in oxidant levels, inflammatory cytokines, blood glucose concentration, and systolic blood pressure. Resveratrol administration to SHR/STZ animals did not impact upon blood glucose levels or haemodynamics suggesting that any cardioprotective effects observed could be attributed to the attenuation of underlying pathological mechanisms.

In the current study, polyphenol therapy was observed to mediate an increase in antioxidant capacity and a reduction in lipid peroxidation. Similar results have been reported following the administration of resveratrol in other* in vivo* and* ex vivo* models and have been attributed to ROS scavenging, downregulation of ROS production, enhancement of natural antioxidant defences, and upregulation of the transcription of endogenous antioxidant genes and cardioprotective molecules [24–27]. In spontaneously hypertensive rats, the administration of resveratrol restored vascular nitric oxide levels and abolished superoxide generation within aortic tissue [28]. Similarly, in isolated myocytes and isolated whole hearts exposed to ischemia/reperfusion injury, the addition of resveratrol inhibited the production of superoxide and upregulated NOS activity [29]. Likewise, in MCF-7 cells, 48 hours of resveratrol treatment reduced the production of ROS and upregulated the expression of antioxidant genes as a result of increased activation of the phosphatase and tensin homolog pathway [26]. Finally, Chan et al. [5] have reported that resveratrol administration to DOCA-salt hypertensive rats led to a reduction in oxidative stress and lipid peroxidation and that these changes conferred improvements in cardiovascular function. These improvements include positive changes in action potential prolongation, left ventricular function, and remodelling and vascular reactivity [5]. Within the current study, increases in nitric oxide coupled with reductions in malondialdehyde prevented the development of vascular and left ventricular dysfunction.

In addition to antioxidant actions, resveratrol administration to SHR/STZ animals mediated moderate anti-inflammatory effects via the reduction of circulating IL-1*β* levels; however, no significant reductions in IL-6 were observed in the current study. Previous reports indicate that the administration of 5 mg/kg of resveratrol to STZ-nicotinamide-induced diabetic rats elicited an anti-inflammatory response by reducing the plasma concentrations of TNF-*α*, IL-1*β*, and IL-6 [30]. Similarly, resveratrol acted in a dose dependent manner to inhibit increased mRNA expression of transcription factors and the production of IL-1*β* and IL-6 in cultured murine microglia and astrocytes [31]. Specifically, inhibition of the Akt/NF*κβ* pathway has been postulated to be associated with the anti-inflammatory effects of resveratrol [32], suggesting resveratrol is able to mediate direct effects on the inflammatory cascade. However, studies in human M1 and M2 macrophages challenged with an autoxidation product of cholesterol also demonstrated that resveratrol is also able to prevent the switching of cells from an anti-inflammatory state to a proinflammatory phenotype [33], thus indicating that resveratrol could mediate protective effects in metabolic disorders and animal models by preventing the recruitment of inflammatory cells as well. Additionally, it is possible that the inability of resveratrol to significantly reduce circulating IL-6 within SHR/STZ animals may be dependent on the dose utilised and that a higher concentration would mediate more extensive anti-inflammatory effects similar to those reported by Buttari et al. [33], Lu et al. [31], and Palsamy and Subramanian [30].

In the current study, aortic tissue isolated from SHR/STZ animals had impaired adrenoceptor-mediated contractile and endothelium-dependent and endothelium-independent relaxation responses, whilst mesenteric arteries had an increase in the magnitude of noradrenaline-induced contractions. Impaired vascular function has previously been reported following the development of both diabetes and hypertension and is attributed to oxidative stress- and inflammatory-mediated changes to endothelial and vascular smooth muscle cells [5, 34–37]. Increased responsiveness of the diabetic vasculature to adrenergic-mediated contractions has also been associated with ineffective release of basal nitric oxide [34, 38]. Consistent with these reports and with our assessment of biochemical markers, the physiological improvements within the SHR/STZ + Res vasculature observed in the current study could be attributed to attenuation of oxidative stress and inflammation. Similar improvements in vasoactive responses to noradrenaline, acetylcholine, and sodium nitroprusside have been reported following the reduction of oxidative stress and lipid peroxidation by resveratrol (1 mg/kg) in aortas isolated from DOCA-salt hypertensive animals [5]. Likewise the administration of resveratrol (20 mg/kg) to homozygote type 2 diabetic mice improved endothelium-dependent relaxation by inhibiting superoxide production and NAD(P)H activation [39]. Therefore, the maintained vascular function noted in SHR/STZ + Res animals within this study has been attributed to the prevention of cellular remodelling by increasing nitric oxide availability and reducing the accumulation of lipid peroxidation by-products and inflammatory cytokines. These findings are also supported by the fact that resveratrol therapy did not alter the background hypertension or hyperglycaemia in this model, yet vascular function was improved. Notably, the administration of resveratrol to WKY animals reduced the endothelium-dependent and endothelium-independent relaxation responses within mesenteric arteries and this observation will require further investigation to elucidate mechanisms.

Resveratrol administration to SHR/STZ animals was also able to prevent the development of cardiovascular dysfunction by improving action potential duration and left ventricular compliance and function. Similar changes in myocardial function have been observed in DOCA-salt rats administered with resveratrol (1 mg/kg) for a period of four weeks and have been attributed to the attenuation of anti-inflammatory, oxidative stress, and antifibrotic pathways [5]. Chan et al. [5] also reported that the administration of resveratrol partially reduced ventricular hypertrophy, a protective effect not observed in the current study. In this manner, it is possible that the dose alone is not the sole factor determining resveratrol's antioxidant and anti-inflammatory effects, as Chan et al. [5] administered half the dose utilised in the current study; rather the animal model utilised and the magnitude of oxidative stress and inflammation are also significant. The antioxidant effects mediated by resveratrol in the current study prevented the development of myocardial dysfunction within the hearts isolated from SHR/STZ animals, thus suggesting the poignant role oxidative stress elicits in this pathological cascade.

Outcomes from the current study indicate that the administration of resveratrol to SHR/STZ animals exhibited antioxidant and moderate anti-inflammatory effects that conferred improvements in vasculature responsiveness and myocardial pump function and electrophysiology. These results indicate that, in a genetic model of hypertension superimposed with type I diabetes, imbalances in redox and inflammatory status are key components in the pathological cascade and that the use of antioxidant and anti-inflammatory compounds can prevent the onset of cardiovascular dysfunction.

## Figures and Tables

**Table 1:** Physiological and biochemical parameters following resveratrol administration to control (WKY + THC) and diabetic rats (STZ + THC).

Parameter	WKY	WKY + Res	SHR/STZ	SHR/STZ + Res
Systolic blood pressure (mmHg)	149 ± 11	157 ± 6	223 ± 9	215 ± 9
Heart rate (BMP)	439 ± 4	413 ± 12	409 ± 9	389 ± 11
Left ventricular mass (mg/g body mass)	2.3 ± 0.03	2.3 ± 0.04	2.8 ± 0.07	2.7 ± 0.07
Right ventricular mass (mg/g body mass)	0.5 ± 0.02	0.5 ± 0.04	0.5 ± 0.02	0.5 ± 0.02
Kidney mass (mg/g body mass)	6.1 ± 0.1	5.8 ± 0.1	9.6 ± 0.2^#^	9.0 ± 0.4^#^
Blood glucose (mmol/L)	10.4 ± 0.7	9.8 ± 0.7	32.8 ± 1.5	29.9 ± 1.1
Serum nitric oxide (*μ*mol)	6.1 ± 0.5	7.1 ± 0.8	4.2 ± 0.3	5.7 ± 0.3
Serum malondialdehyde (*μ*mol)	62.2 ± 2.7	60.2 ± 2.5	127.8 ± 5.7	108.7 ± 4.7
Serum interleukin-1*β* (ng/mL)	4.8 ± 0.1	4.8 ± 0.1	6.1 ± 0.3	5.2 ± 0.2
Serum interleukin-6 (ng/mL)	3.1 ± 0.1	3.4 ± 0.1	3.4 ± 0.1	3.5 ± 0.1

Values are presented as mean ± SEM. ^#^ *P* < 0.05 versus WKY.

**Table 2:** Left ventricular pump function and microelectrode parameters following resveratrol administration to control (WKY + Res) and diabetic rats (STZ + Res).

Parameter	WKY	WKY + Res	SHR/STZ	SHR/STZ + Res
Diastolic stiffness	23.7 ± 0.7	22.9 ± 0.9	26.1 ± 0.9^#^	21.3 ± 0.9^†^
+dP/dT_max_ (mmHg/sec)	2025 ± 115	2256 ± 138	1488 ± 211^#^	2200 ± 116^†^
−dP/dT_max_ (mmHg/sec)	−1206 ± 97	−1394 ± 118	−887 ± 131^#^	−1440 ± 82^†^
Developed pressure (mmHg)	112.6 ± 5.6	124.0 ± 7.4	84.6 ± 11.5^#^	103.8 ± 8.6
End systolic pressure (mmHg)	120.0 ± 6.2	134.1 ± 7.2	96.2 ± 11.7	114.8 ± 9.1
Action potential duration at 20% of repolarisation (msec)	18.7 ± 0.9	20.6 ± 0.7	35.6 ± 1.8^#^	29.4 ± 1.9^†^
Action potential duration at 50% of repolarisation (msec)	28.7 ± 1.2	31.2 ± 1.4	66.3 ± 2.4^#^	46.8 ± 4.3^†^
Action potential duration at 90% of repolarisation (msec)	58.8 ± 3.2	66.7 ± 6.1	123.6 ± 9.2^#^	89.4 ± 6.6^†^
Resting membrane potential (mV)	−65.0 ± 2.1	−67.1 ± 1.7	−59.1 ± 2.0	−61.1 ± 3.0
Force of contraction (mN)	2.6 ± 0.7	2.7 ± 0.4	1.7 ± 0.3	1.8 ± 0.3

Values are presented as mean ± SEM. ^#^ *P* < 0.05 versus WKY, ^†^ *P* < 0.05 versus SHR/STZ.
